# Photocarcinogenesis of the skin: Current status and future trends

**DOI:** 10.1002/kjm2.12946

**Published:** 2025-02-05

**Authors:** Ting‐Ting Yang, Cheng‐Che E. Lan

**Affiliations:** ^1^ Department of Dermatology, Kaohsiung Medical University Gangshan Hospital, Kaohsiung Medical University Hospital Kaohsiung Medical University Kaohsiung Taiwan; ^2^ Department of Dermatology, Kaohsiung Medical University Hospital Kaohsiung Medical University Kaohsiung Taiwan

**Keywords:** skin cancer, ultraviolet radiation, visible light

## Abstract

Solar radiation is essential for life on Earth but is also a major contributor to skin carcinogenesis. Solar radiation, particularly ultraviolet (UV) B (280–320 nm) and UVA (320–400 nm), induces photocarcinogenesis via various pathways. UV light can directly cause DNA damage, resulting in genetic mutations if not repaired correctly. UV light can also induce photocarcinogenesis by generating reactive oxygen species, inducing immunosuppression and inflammation. Recently, visible light (400–760 nm) has been shown to contribute to photocarcinogenesis by activating oxidative pathways. In addition to the irradiation dose (fluence, J/m^2^), UVB irradiance (W/m^2^) is also considered a factor influencing photocarcinogenesis. In this review, we summarize the mechanisms of photocarcinogenesis and provide strategies to prevent skin cancer.

## 1 INTRODUCTION

Solar radiation is a substantial energy source for life on Earth and influences many aspects of the human body. The spectrum of solar radiation reaching the surface of the Earth ranges from 5.5% ultraviolet (UV) light (100–400 nm), 43% visible light (VL) (400–760 nm) to 51.5% infrared (>760 nm).^1^ The UV spectrum can be further divided into UVA (320–400 nm), UVB (280–320 nm), and UVC (100–280 nm). As almost all radiations in the UVC spectrum are filtered out by the ozone layer, most UV radiations reaching the skin fall within the UVB (5%) and UVA (95%) spectrums.^2^

**FIGURE 1:**
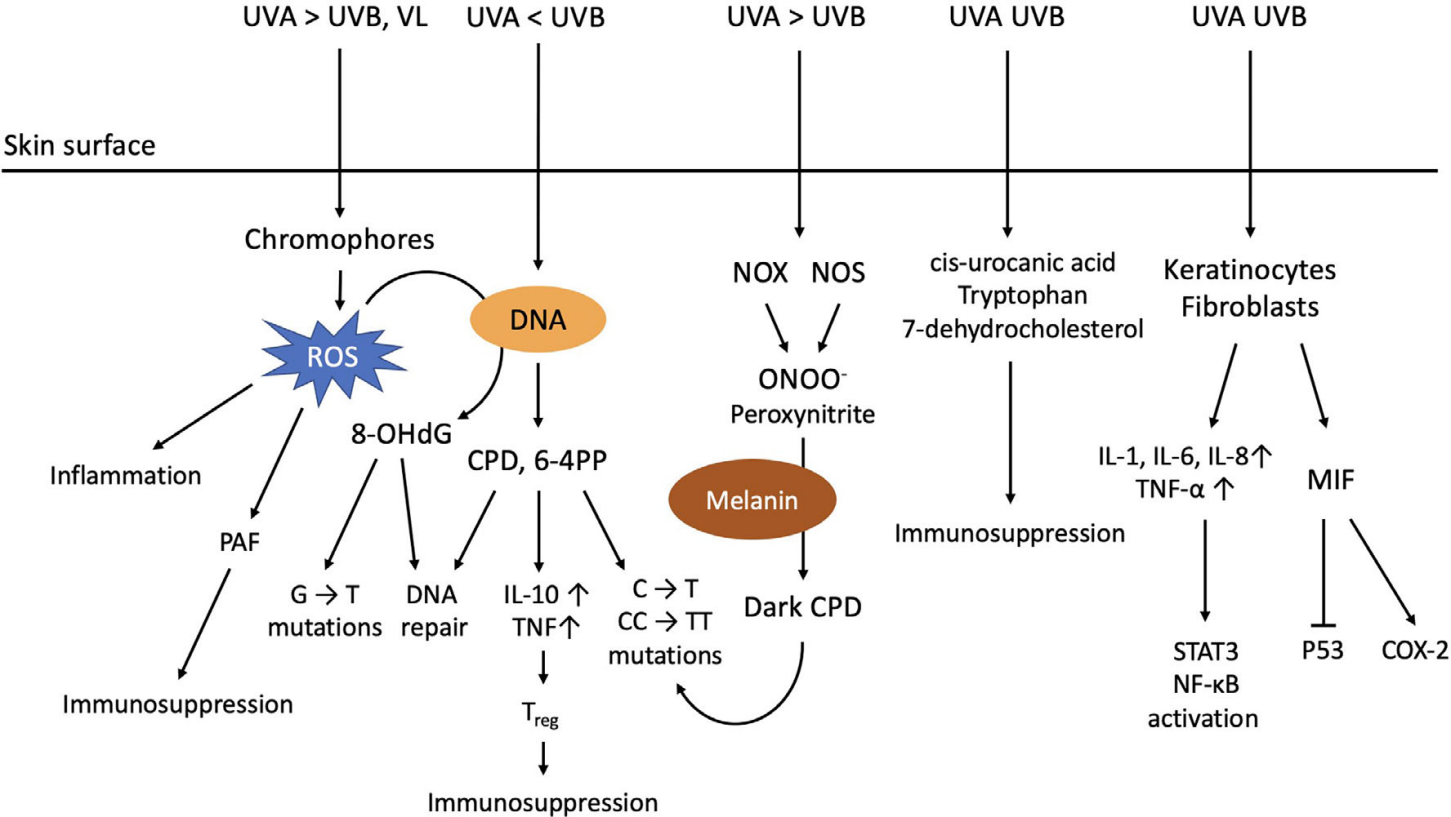
Summary of cutaneous photocarcinogenesis mechanisms. Ultraviolet A (UVA) and ultraviolet B (UVB) induce photocarcinogenesis by triggering DNA damage, oxidative stress, immunosuppression, and inflammatory pathway activation. Visible light (VL) also contributes to photocarcinogenesis by inducing oxidative stress. CPD, cyclobutane‐pyrimidine dimer; MIF, macrophage inhibitory factor; NOS, nitric oxide synthase; NOX, nicotinamide adenine dinucleotide phosphate oxidase; PAF, platelet‐activating factor; 6‐4PP, 6–4 photoproducts.

Compared to other organs, the outermost layer of the human body, the skin, is constantly affected by solar radiation. Although UV radiation is only a small proportion of solar radiation, its interaction with the skin is essential in maintaining human health, such as vitamin D synthesis and β‐endorphin production.^3^, ^4^ However, UV radiation has also been shown to contribute substantially to skin cancer development, including melanoma and nonmelanoma skin cancers, by inducing DNA damage, oxidative stress, and immunosuppression.^5^, ^6^ VL has been found to contribute to skin cancer development by activating oxidative pathways in keratinocytes and melanocytes.^7^ In this article, we aimed to summarize the mechanisms of solar radiation‐induced cutaneous photocarcinogenesis. The mechanisms of photocarcinogenesis discussed in this review are illustrated in Figure 1.

## 2 DIRECT EFFECT OF UV RADIATION ON CELLULAR DNA

### 2.1 DNA damage

DNA damage is a critical step in cancer development. UV radiation causes direct damage to the DNA of skin cells. Compared with UVA, UVB is considered more carcinogenic because it is more efficiently absorbed by DNA.^8^ The main photoproducts induced by UV radiation are cyclobutane‐pyrimidine dimers (CPDs), 6‐4 photoproducts (6‐4PPs), and their Dewar valence isomers.^8^, ^9^ Although 6‐4PPs have higher mutagenicity, CPDs are the most abundant UV photoproducts as they are repaired more slowly than 6‐4PPs.^8^ In addition to these photoproducts, UV radiation can induce single‐ and double‐strand breaks, leading to cellular damage and genetic aberration.^8^

CPDs are ring structures formed by the covalent linkage of the C5 and C6 carbon atoms of two adjacent pyrimidine bases. In 6‐4PPs, the C6 carbon of one pyrimidine base is linked to the C4 carbon of the neighboring pyrimidine base. CPDs and 6‐4PPs can be formed from any pyrimidine combination, including thymine–thymine (TT), cytosine–thymine (CT), and cytosine–cytosine (CC) dimers. Failure to repair these photoproducts leads to cytosine to thymine (C → T) DNA base transitions and the tandem double CC → TT mutation, the UV signature mutations. Other less common photoproducts are adenine dimers, adenine–thymine photoadducts, Porschke photoproducts, 4,6‐diamino‐5‐guanidinopyrimidine, and 8‐(5‐aminoimidazol‐4‐yl)adenine.^8^

### 2.2 Clone expansion

Because chronic sun exposure in normal‐looking skin has the same mutations as in malignant skin lesions, DNA mutations are not the only factors leading to cancer development.^10^ Clonal expansion of cells with DNA damage is a pivotal step in tumor formation, and cellular mutation load correlates with tumor burden.^10^ Mutations in several driver genes, including TP53, NOTCH1, NOTCH2, PPMID, and FAT1, have been identified as leading contributors to clonal expansion in squamous cell carcinomas.^10^ Mutations in the Hedgehog pathway, PTCH1, SMO, and TP53 are the major driver mutations in basal cell carcinomas.^11^ BRAF, RAS, NF1, and TERT are the most common driver mutations leading to tumor progression in cutaneous melanomas.^12^, ^13^

### 2.3 DNA repair

DNA damage caused by UV radiation can be repaired via several cellular DNA repair pathways to prevent the accumulation of DNA mutations. CPD and 6‐4PPs are predominantly repaired via the nucleotide excision repair (NER) pathway, which involves DNA damage recognition, excision of DNA lesions, and repair synthesis.^14^ The importance of DNA repair mechanisms is best demonstrated in diseases impairing DNA repair pathways. Xeroderma pigmentosum is a disease characterized by protein mutations in the NER pathway, resulting in increased photosensitization, premature photoaging, and a more than 1000‐fold increased risk of skin cancer.^15^, ^16^ Other diseases resulting from NER pathway impairment and increased risk of skin cancer include Cockayne syndrome, trichothiodystrophy, and UV‐sensitive syndrome.^17^

## 3 THE INDIRECT EFFECT OF UV RADIATION ON CELLULAR DNA

### 3.1 Reactive oxygen species

Reactive oxygen species (ROS) are oxygen‐containing, highly reactive molecules that include the superoxide anion ($O_{2}^{•-}$), singlet oxygen (^1^O_2_), hydroxyl radical (•OH), hypochlorous acid (HOCl), and hydrogen peroxide (H_2_O_2_).^18^ ROS are involved in many critical physiological processes, including cell signaling and defense against pathogens. However, elevated ROS levels contribute to cancer development by causing DNA damage.^18^ UVA and, to a lesser extent, UVB radiation can trigger ROS production in the skin by stimulating several cell chromophores.

UVB radiation can induce the superoxide anion ($O_{2}^{•-}$), hydroxyl radical (•OH), and hydrogen peroxide (H_2_O_2_) by activating cellular enzymes nicotinamide adenine dinucleotide phosphate (NADPH) oxidase (NOX), cyclooxygenase, and catalase.^19^ UVB can cause a bystander effect in adjacent nonirradiated cells, inducing superoxide anion ($O_{2}^{•-}$).^20^ Similar to UVB radiation, UVA can induce ROS production by activating cellular enzymes and triggering bystander effects.^20^ UVA induces ROS through two main mechanisms in the photoexcitation of chromophores (e.g., porphyrins, heme, melanin, pterins, and flavins) in the cell.^20^, ^21^ The first mechanism (type I photosensitization) involves electron transfer reactions of molecules leading to the production of superoxide anion ($O_{2}^{•-}$) and hydroxyl radical (•OH).^20^ The second mechanism (type II photosensitization) induces singlet oxygen (^1^O_2_) via energy transfer to molecular oxygen (O_2_).^20^

The accumulation of cellular ROS causes DNA damage by oxidizing nitrogen bases and, less commonly, through single‐ and double‐stranded DNA breaks.^21^ The oxidative DNA damage caused by ROS predominantly involves guanine bases.^21^ The most common and best‐studied ROS‐induced DNA damage is 8‐hydroxy‐2′‐deoxyguanosine (8‐OHdG) formation.^21^ Unrepaired 8‐OHdG is highly mutagenic and leads to transitions from guanine to thymine (G → T).^21^ In addition to damaging DNA, ROS can cause damage by oxidizing cellular lipids and proteins.^19^

### 3.2 UV‐induced inflammation

Chronic inflammation increases the risk of skin cancer development.^22^, ^23^ UV‐induced DNA damage and ROS production trigger inflammation. The immune system recognizes DNA lesions and ROS‐induced damaged cellular structures and triggers inflammatory reactions.^23^ ROS‐induced oxidative stress also activates chronic inflammation.^23^ In addition, UV radiation can trigger inflammation by activating keratinocytes and fibroblasts at higher doses.^24^ Activated keratinocytes and fibroblasts release proinflammatory signals and recruit immune cells, amplifying the inflammatory process. UVB induces the production of proinflammatory cytokines IL‐1α and IL‐1β, and UVA and UVB may induce TNF‐α, IL‐6, and IL‐8.^23^, ^24^ Macrophage migration inhibitory factor (MIF) is a proinflammatory cytokine produced by keratinocytes upon UVB irradiation.^24^ MIF triggers skin inflammation by inhibiting p53 and activating cyclooxygenase‐2.^24^

Of the inflammatory signals released by UV radiation, TNF‐α, IL‐1, IL‐6, and IL‐8 are activators of the STAT3 (signal transducer and activator of transcription 3) and NF‐κB signaling pathways.^23^ STAT3 and NF‐κB play important roles in regulating malignant cell apoptosis, angiogenesis, invasiveness, and tumor inflammatory microenvironment.^25^

### 3.3 Dark cyclobutene pyrimidine dimers

The effect of UV radiation on the skin persisted even after discontinuing UV exposure. Continuous CPD formation was observed up to 2–4 hours after cessation of UVA or UVB irradiation, and these CPDs are termed “dark CPDs.”^26^, ^27^ Dark CPDs are formed by the reaction between UV‐induced ROS and melanin in melanocytes and keratinocytes. UV‐induced ROS stimulate NOX and nitric oxide synthase (NOS).^28^ Subsequently, NOX and NOS generate peroxynitrite (ONOO^−^), a highly reactive oxidant.^28^ Peroxynitrite further reacts with melanin to generate its excited triplet state melanin‐carbonyls.^28^ These melanin radicals have an energy approximately equivalent to that of UV photons and can trigger DNA CPDs.^28^ Compared to eumelanin, pheomelanin produces 3–5 times more CPDs, enhancing its role in skin cancer development.^29^

## 4 UV RADIATION AND IMMUNOSUPPRESSION

Immunosurveillance of malignant cells is an important mechanism that inhibits tumor development.^30^ The effect of immunosuppression on cancer progression is observed under congenital and acquired immunodeficiencies.^31^, ^32^ The relative risk of developing nonmelanoma skin cancers in organ transplant recipients has been reported to be more than 100‐fold higher compared with the normal population.^33^

Early studies have shown that UV‐induced immunosuppression inhibits contact hypersensitivity reactions after UV radiation and hampers the immune response toward skin cancer.^34^, ^35^ Several pathways are involved in UV‐induced immunosuppression. One common outcome of the immunosuppressive pathways is a decrease in epidermal Langerhans cells (LCs) and an increase in regulatory *T* cells (*T*_reg_) in the epidermis. *T*_reg_ cells are a subset of CD4+ T cells with immunosuppressive properties essential for maintaining self‐tolerance.^36^ However, *T*_reg_ cells also inhibit the immunosurveillance of tumor cells, leading to immune evasion of malignant cells and, consequently, to tumor development and progression.^36^

UV radiation‐induced DNA damage triggers UV‐induced immunosuppression. CPD and 6‐4PPs contribute to UV‐induced immunosuppression, although CPD may play a more considerable role.^34^, ^37^ CPD formation in keratinocytes and LCs induces IL‐10 and TNF production, leading to decreased antigen‐presenting ability of LCs and induction of *T*_reg_ cells.^34^ ROS formation is another important factor leading to UV‐induced immunosuppression, and ROS inhibition reduces UV‐induced immunosuppression.^37^ ROS generated by UV radiation induce immunosuppression by increasing platelet‐activating factor production and inducing DNA damage.^37^ *Cis*‐urocanic acid is formed by the isomerization of *trans*‐urocanic acid upon absorption of UV radiation. *Cis*‐urocanic acid induces immunosuppression by binding to the serotonin receptor 5‐HT_2A_ in keratinocytes, mast cells, and antigen‐presenting cells.^35^ Tryptophan is a chromophore of UV radiation. After absorbing UV radiation, tryptophan activates the aryl hydrocarbon receptor, which stimulates cyclooxygenase‐2 to produce suppressive prostaglandins, such as PGE_2_.^35^ UV radiation converts 7‐dehydrocholesterol to pre‐vitamin D_3_, further converted into its biologically active form 1,25‐dihydroxyvitamin D_3_ (1,25(OH)_2_D_3_). 1,25(OH)_2_D_3_ contributes to immunosuppression by inducing dendritic cell maturation and *T*_reg_ population expansion.^35^

## 5 INFLUENCE OF UV IRRADIANCE ON PHOTOCARCINOGENESIS

The incidence of skin tumor formation depends on UV exposure in a dose‐dependent manner.^38^, ^39^ The relationship between UV dose and cancer burden is stronger for squamous cell carcinomas than for basal cell carcinomas and melanomas, in which irradiation patterns also play an essential role in carcinogenesis.^38^ However, in addition to the total irradiation dose and irradiation pattern, irradiance has been identified as an important factor influencing photocarcinogenesis.

**FIGURE 2:**
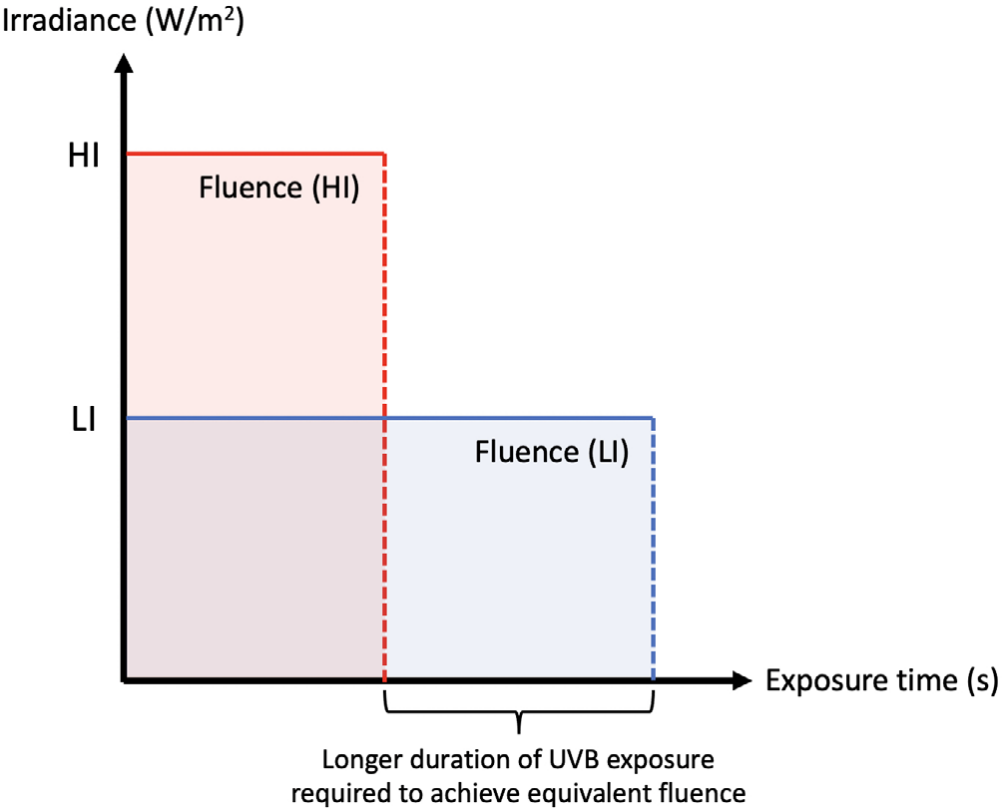
Fluence (J/m^2^) is the product of irradiance (W/m^2^) and total irradiation time (s). Prolonged exposure time is required to achieve equivalent fluence for low irradiance ultraviolet B (LIUVB) irradiation (blue area) compared to high irradiance ultraviolet B (HIUVB) irradiation (red area).

**FIGURE 3:**
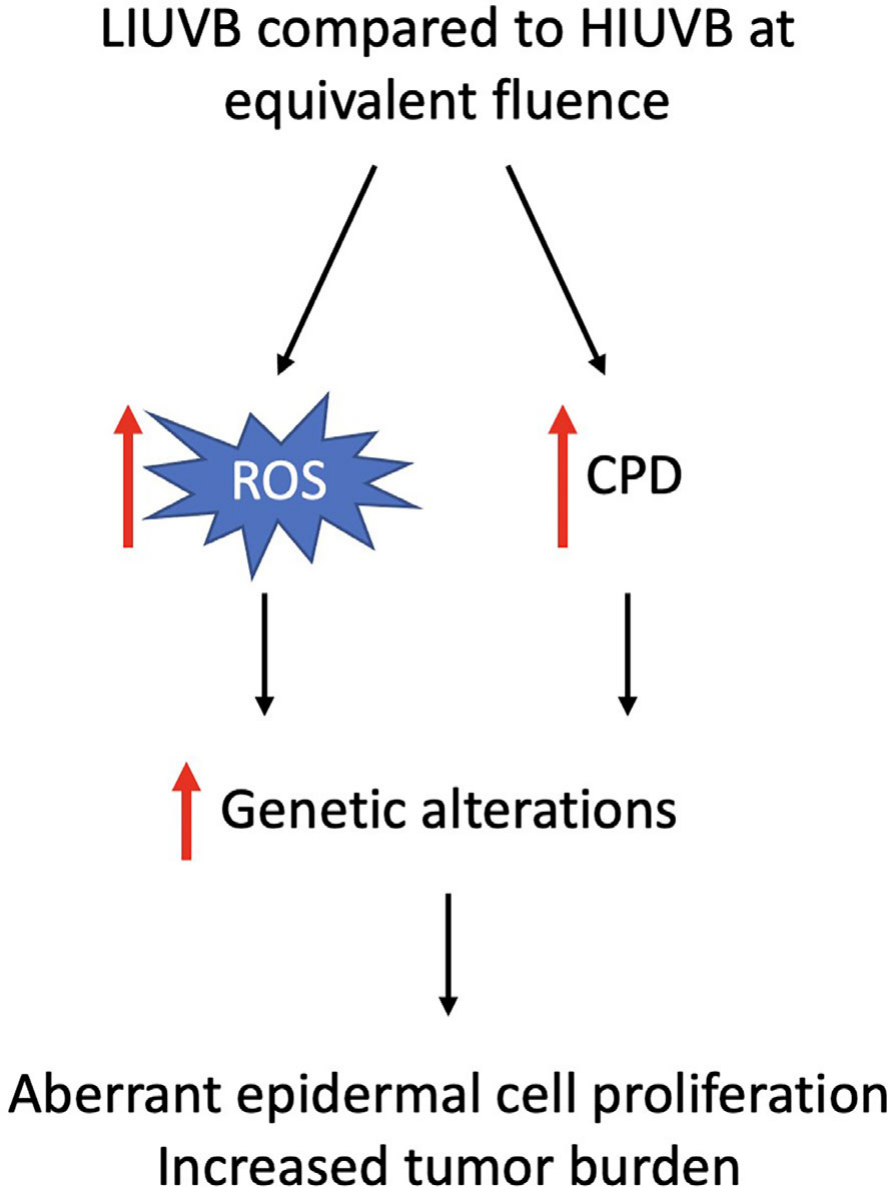
Low irradiance ultraviolet B (LIUVB) induces more aberrant epidermal cell proliferation and tumor burden than high irradiance ultraviolet B (HIUVB) by inducing more reactive oxygen species (ROS) and cyclobutane pyrimidine dimer (CPD) formation.

The total dose (fluence, J/m^2^) is the product of irradiance (photon density, W/m^2^) and exposure time (s) (Figure 2). In 1923, Bunsen and Roscoe proposed the reciprocal law, which states that photochemical reaction is the same regardless of the irradiance or irradiation time as long as the fluence is equal.^40^ A classic example of this law is skin erythema or sunburn. When a certain threshold of UVB radiation fluence is reached, skin erythema develops regardless of the UVB irradiance.^41^ However, UVB‐induced photocarcinogenesis does not follow this rule. Low‐irradiance UVB (LIUVB) increases tumor formation and aberrant epidermal proliferation by inducing more DNA damage and ROS formation compared with high‐irradiance UVB (HIUVB) at an equivalent fluence. In animal studies, hairless mice irradiated with LIUVB showed increased epidermal proliferation and tumor burden when irradiated with UVB at an equivalent fluence, compared with their HIUVB counterparts.^41^, ^42^ Keratinocytes irradiated with LIUVB harbored higher CPD levels and increased numbers of cells entering the S phase compared with HIUVB‐treated keratinocytes.^43^ Moreover, LIUVB generates higher levels of ROS and 8‐OHdG than those of HIUVB in cultured keratinocytes and murine epidermis at equivalent fluences.^41^, ^44^ These results indicate that, in addition to total UVB dose, UVB irradiance is a considerable factor contributing to skin photocarcinogenesis (Figure 3).

## 6 BEYOND THE UV SPECTRUM: EFFECT OF VL ON PHOTOCARCINOGENESIS

Compared with UV radiation, a much larger proportion of solar radiation reaching the skin is VL (400–760 nm) (5.5% vs. 43%). VL induces skin pigmentation and photoaging.^45^ Radiation in the VL spectrum has also been shown to play a role in photocarcinogenesis by causing cellular oxidative stress and DNA damage.

Cellular chromophores, including melanin, porphyrins, flavins, and lipofuscin, absorb energy from the VL and generate free radicals that cause DNA damage.^7^, ^46^, ^47^ Owing to their abundance in solar radiation, approximately 50% of the free radicals generated in the skin are induced by VL.^7^ In cultured Chinese hamster cells, DNA base modifications, including 8‐OHdG and formamidopyrimidines, can be induced using radiation at wavelengths up to 490 nm.^48^ Lipofuscin is a pigmented granule composed of oxidized proteins, degraded lipids, and a small amount of metal that accumulates as the cell ages.^49^ Upon VL irradiation, the lipofuscin granules become photoexcited and generate ROS, including triplet species and singlet oxygen.^50^ VL has also been shown to cause DNA strand breaks in synergy with UVA.^50^

Within the VL spectrum, blue light (380–500 nm) causes the greatest damage to the skin. In cellular studies, VL in the violet to blue spectrum induces the most DNA damage, oxidative stress, and damage to mitochondria and lysosomes.^51^ Hiramoto et al.^52^ demonstrated that blue light, but not green or red light, induced microscopic skin cancer lesions in murine models after daily irradiation for 1 year. Indicators of cellular proliferation, including ki‐67 and cyclin D1, were also higher in blue light‐irradiated murine skin than in green or red light‐irradiated subjects.^52^

The current evidence suggests that VL is a potential contributor to photocarcinogenesis, especially in synergy with UV radiation. However, further studies are needed to confirm whether VL can induce skin cancer independently. Recently, a decrease in melanoma tumor size following green light irradiation was demonstrated in animal models, suggesting a protective role of ROS from melanin photosensitized by VL in tumor progression.^53^

## 7 PREVENTION OF PHOTOCARCINOGENESIS AND PHOTOPROTECTION

Several strategies have been developed to protect skin from the harmful effects of solar radiation. One strategy is to reduce the number of photons reaching the skin by applying sunscreen. Sunscreen UV filters can be either organic or inorganic. Organic filters contain compounds that convert photon energy into other forms of energy, including heat. In contrast, inorganic UV filters reduce UV photons by deflecting and scattering UV radiation. Regular sunscreen application has been shown in clinical trials to prevent melanoma and nonmelanoma skin cancer occurrence.^54^, ^55^ However, despite the increasing proportion of people using sunscreen, the incidence of skin cancer continues to rise.^56^, ^57^ One possible explanation for this is the increased rate of sunburn after sunscreen application. Sunscreen users are more likely to have prolonged sun exposure and experience sunburn.^58^, ^59^ UV irradiation may also play a role in this process. As mentioned, LIUVB induces a greater tumor burden and increases aberrant epidermal proliferation compared with HIUVB at equivalent fluences in animal studies.^41^, ^42^, ^44^ Applying sunscreen is similar to lowering the UV irradiance received by the skin. The negative effect of UVB on photocarcinogenesis may be more severe in sunscreen‐applied skin (LIUVB) than in ordinary skin (HIUVB) as the total dose of UVB entering the skin reaches a certain threshold (e.g., sunburn). In addition to UV radiation, VL has been identified as an important factor in photocarcinogenesis. Conventional organic and inorganic UV filters provide limited protection against VL. Currently, only tinted sunscreens containing titanium dioxide and iron oxide provide good protection against UVA1 (370–400 nm) and VL.^60^, ^61^

Another strategy to prevent photocarcinogenesis is incorporating antioxidants to target ROS induced by solar radiation.^62^ Topical and oral vitamin C (L‐ascorbic acid) and E (tocopherol) reduce solar oxidative damage by reducing ROS.^63^ Oral carotenoids, especially β‐carotene, have potent antioxidant properties and have been used as photoprotective agents in erythropoietic protoporphyria.^64^ Polyphenols, including flavonoids and nonflavonoids, are effective in reducing UV‐induced oxidative stress and have anti‐inflammation properties.^65^ Polyphenols have been demonstrated to inhibit photocarcinogenesis in animal models.^65^ However, there is currently insufficient evidence that antioxidants alone can prevent photocarcinogenesis, and they should be used with topical sunscreens.

UV‐induced immunosuppression contributes to photocarcinogenesis. Current sunscreens provide some effects to prevent UV‐induced immunosuppression, especially when adequate UVA protection is provided.^66^ Another strategy to reduce UV‐induced immunosuppression while enhancing DNA repair is chemoprevention using nicotinamide (the amide form of vitamin B3). Oral and topical nicotinamide has been shown to prevent nonmelanoma skin cancer development by reducing the effect of UV‐induced immunosuppression and enhancing DNA repair.^67^, ^68^, ^69^, ^70^ Topical green tea polyphenol has also been shown to reduce UVB‐induced immunosuppression in animal models.^71^

As the total mutation load correlates with the skin tumor burden, it has become a target for skin cancer prevention. Reducing the total mutational load in solar‐damaged skin by ablative laser or dermabrasion prevents the formation of squamous and basal cell carcinomas.^72^, ^73^

## 8 CONCLUSIONS

Photocarcinogenesis involves an interplay of DNA photoproducts, DNA repair mechanisms, ROS formation, skin inflammation, and immunosuppression. Understanding photocarcinogenesis mechanisms is essential for developing preventive strategies. Efficient photoprotection strategies should address these mechanisms and include solar radiation beyond the UV spectrum.

## CONFLICT OF INTEREST STATEMENT

The authors declare no conflicts of interest.

## Data Availability

Data sharing is not applicable to this article as no new data were created or analyzed in this study.
